# Association between triglyceride-glucose index and stages of T2DM: potential indirect effects of inflammatory indicators

**DOI:** 10.3389/fendo.2026.1931822

**Published:** 2026-10-02

**Authors:** Jiayi Tian, Haitao Li, Mingyi Li, Qingning Chang, Xianglong Liu, Yuhao Zhou, Yu Zhao, Feng Li

**Affiliations:** 1 School of Public Health, Ningxia Key Laboratory of Environmental Factors and Chronic Disease Control, Ningxia Medical University, Yinchuan, China; 2 Center of Laboratory Medicine, General Hospital of Ningxia Medical University, Yinchuan, Ningxia, China

**Keywords:** inflammatory indicators, mediating effect, triglyceride-glucose index, type 2 diabetes mellitus, type 2 diabetes mellitus staging

## Abstract

**Background:**

The triglyceride-glucose (TyG) index is a reliable surrogate marker of insulin resistance and has been closely linked to type 2 diabetes mellitus (T2DM) risk. However, it remains unclear whether the TyG index is nonlinearly associated with cross-sectional glycemic status categories, from normal glucose to T2DM, and whether inflammatory indicators may partly account for this association. We therefore aimed to examine this nonlinear association and to estimate potential indirect associations through inflammatory indicators.

**Methods:**

A retrospective analysis of cross-sectional data was conducted, including 1,117 participants. Generalized additive models (GAM) combined with restricted cubic spline (RCS) regression were used to examine the nonlinear association between the TyG index and stages of glucose metabolism. Stage specificity analyses were performed across normal glucose, impaired fasting glucose (IFG), impaired glucose tolerance (IGT), combined IFG and IGT, and T2DM. In exploratory cross-sectional indirect association analyses, inflammatory markers accounted for part of the cross-sectional association between the TyG index and glycemic outcomes.

**Results:**

A significant nonlinear relationship was identified between the TyG index and T2DM (*P* < 0.05), with an inflection point at TyG = 9.14. The TyG index showed a progressively increasing trend across advancing disease stages. In exploratory cross-sectional analyses of potential indirect associations, only MLDLR and NLDLR exhibited significant statistical indirect associations linking the TyG index to 2-hour postprandial plasma glucose (2hPG). However, because all variables were measured concurrently, causal mediation cannot be inferred. These exploratory findings may warrant further investigation in longitudinal studies, particularly across early stages of dysglycemia.

**Conclusions:**

The TyG index was nonlinearly associated with cross-sectional glycemic categories. Exploratory cross-sectional mediation analyses further revealed statistically significant indirect associations through MLDLR and NLDLR in the TyG index–2hPG relationship. These observations suggest that inflammatory markers may be related to glycemic deterioration primarily via postprandial-related pathways in early dysglycemia, although causal inference cannot be drawn from cross-sectional data. These findings are hypothesis-generating and support the prospective evaluation of the TyG index for risk stratification in primary-care settings.

## 1 Introduction

Diabetes is a chronic metabolic disorder characterized by persistently high levels of blood glucose due to a deficiency in insulin secretion or a dysfunction of its biological effects (1). It is estimated that the global number of people with diabetes will rise from over 500 million (approximately 6% prevalence) to more than 1 billion (approximately 10%) by 2050, among which the prevalence rate of type 2 diabetes mellitus (T2DM) will account for more than 90% (2). Given the progressive onset of T2DM, prevention and early diagnosis are of vital importance (3).

In 2019, The Lancet Diabetes & Endocrinology published a classic study on diabetes prevention in Daqing, China. If prediabetes is not intervened in, about 7% of individuals with prediabetes will develop T2DM each year, and the cumulative incidence of T2DM among prediabetic individuals after 30 years is as high as 95.9% (4). According to a report in the New England Journal of Medicine in 2002, intensive lifestyle intervention can significantly reduce the incidence of diabetes by 58% over nearly three years (5). Thus, the research hotspot has shifted from clinical diagnosis of diabetes to the critical prediabetic stage.

Insulin resistance is the critical pathophysiological mechanism in the onset and progression of prediabetes. Metabolic disorders mediated by obesity are the most significant risk factor for insulin resistance (6). The TyG index is calculated from fasting blood glucose and fasting triglyceride levels (7). As a simple and readily accessible surrogate marker of insulin resistance, numerous epidemiological studies have confirmed its strong association with diabetes risk. Most studies operationalize diabetes outcomes as a binary variable, distinguishing merely between incidence and non-incidence. For instance, Low et al., in a community-based cohort study, used only fasting plasma glucose ≥ 7.0 mmol/L or use of antihyperglycemic medications as the diabetes endpoint and analyzed it based on the Cox proportional hazards model (8). However, this approach ignores the continuous, stepwise progression from normal glucose metabolism to prediabetes and then to clinical diabetes, and cannot assess the stage-specific impact of the TyG index on transition risk across different stages. Thus, in this study, the TyG index was selected as the independent variable to further explore its association with cross-sectional glycemic status categories.

Obesity may lead to adipose tissue accumulation and hypertrophy, resulting in tissue hypoxia and cellular stress, which in turn trigger the release of various pro-inflammatory cytokines and hormones (9). These substances impair insulin signaling, reduce glucose uptake and utilization efficiency, disrupt normal glucose metabolism, and thereby increase the risk of T2DM (10). Inflammatory indicators represent one key mechanistic pathway (11). Although the association between insulin resistance and chronic low-grade inflammation has been widely confirmed, and studies have shown that an elevated TyG index is significantly correlated with increased levels of various inflammatory markers, including high-sensitivity C-reactive protein (hs-CRP) and interleukin-6 (IL-6), it remains unclear which inflammatory markers may partly explain the association between the TyG index and glycemic outcomes, and whether such indirect associations differ across cross-sectional glycemic categories (12). Although some studies have conducted mediation analyses, these studies were largely limited to specific subgroups, such as pregnant women with gestational diabetes, and examined inflammatory markers as outcomes rather than cross-sectional glycemic categories, from normal glucose to T2DM (13). Accordingly, the present study systematically evaluates 12 routinely available composite inflammatory indicators as potential mediators between the TyG index and 2-hour postprandial glucose across the full spectrum of glycemic stages.

This study conducted a retrospective cross-sectional analysis with the aim of achieving the following goals: firstly, to assess the association between the TyG index and cross-sectional glycemic status categories; secondly, to quantify the possible statistical indirect association through inflammatory indicators in the relationship between the TyG index and 2-hour postprandial plasma glucose (2hPG). By addressing these questions, this study can generate hypothesis-driven evidence for evaluating the TyG index in stage-stratified T2DM risk prediction and offer insights for inflammation-targeted interventional research. It should be emphasized that the glycemic stages examined in this study were defined from concurrent laboratory values in a cross-sectional design. The pathways described below represent the hypothesized natural-history sequence of T2DM used to organize stage-specific comparisons, and do not constitute longitudinal follow-up of individual participants.

## 2 Subjects and methods

### 2.1 Study subjects

This is a retrospective cross-sectional study. All laboratory measurements used to compute the TyG index and to assess inflammatory mediators, fasting plasma glucose, and 2-hour postprandial plasma glucose were obtained from blood specimens collected during a single clinical encounter per participant. No temporally separated repeated measurements or follow-up data were available. Participants were recruited from the General Hospital of Ningxia Medical University between January 2022 and December 2025. The inclusion criterion was completion of a 2hPG test. A total of 1,136 individuals with 2hPG data were initially identified. After excluding 19 duplicate records, 1,117 participants were retained. Among these, 2hPG data were complete for all participants. Missing data were present in other covariates: FPG (88 missing, 7.9%), triglycerides (582 missing, 52.0%), and peripheral blood cell counts (475 missing, 42.5%). Complete data for all covariates were available for only 394 participants (35.3%). (The inclusion and exclusion flowchart can be found in **Supplementary Figure A1** in the supplementary.) Missing values were handled using multiple imputation, and all subsequent statistical analyses were performed on the multiply imputed dataset.

### 2.2 Methods

#### 2.2.1 Definition of the TyG index

The TyG index was calculated using the following formula:

TyG index = ln [fasting TG (mg/dL) × fasting glucose (mg/dL)]/2 (7).

#### 2.2.2 Definition of cross-sectional glycemic categories from normal glucose to T2DM

1. Normal Glucose Tolerance (NGT): Fasting plasma glucose (FPG) is less than 5.6, and the 2hPG is lower than 7.8 millimoles per liter.
2. Impaired Fasting Glucose (IFG): FPG is between 5.6 mmol/L and 6.9 mmol/L (inclusive of 5.6 mmol/L and exclusive of 6.9 mmol/L), or 2hPG is less than 7.8 mmol/L.
3. Impaired Glucose Tolerance (IGT): FPG is less than 5.6 mmol/L, or 2hPG ranges from 7.8 mmol/L to 11.0 mmol/L.
4. Impaired Glucose Tolerance and Impaired Fasting Glucose (IGT + IFG): FPG is between 5.6 mmol/L and 6.9 mmol/L (inclusive of 5.6 mmol/L and exclusive of 6.9 mmol/L), or the 2hPG ranges from 7.8 mmol/L to 11.0 mmol/L.
5. Type 2 Diabetes Mellitus (T2DM): FPG is greater than or equal to 6.9 mmol/L, and the 2hPG is greater than 11.0 mmol/L (14).

These are cross-sectional glycemic categories and are not treated as longitudinally observed transitions.

#### 2.2.3 Definition of the inflammation index

1. AISI (15) = (Neutrophil count × Platelet count × Monocyte count)/Lymphocyte count;
2. SIRI (16) = (Neutrophil count × Monocyte count)/Lymphocyte count;
3. NMLR (17) = (Monocyte count + Neutrophil count)/Lymphocyte count;
4. MLR (18) = (Monocyte count/Lymphocyte count);
5. NLR (19) = (Neutrophil count/Lymphocyte count);
6. SII (20) = (Platelet count × Neutrophil count)/Lymphocyte count;
7. LHR (21) = (Lymphocytes/HDL);
8. LLR (21) = (Lymphocytes * LDL);
9. MHR (22) = (Monocytes/HDL);
10. MLDLR (22) = (Monocytes * LDL);
11. NLDLR (23) = (Neutrophils * LDL);
12. NHDLR (23) = (Neutrophils/HDL).

#### 2.2.4 Covariates

Covariates were selected based on a multivariable conceptual model of factors potentially influencing the TyG index and diabetes diagnosis. The covariates in this study included demographic and health-related variables, such as age (years), sex (male or female), total cholesterol (TC), high-density lipoprotein cholesterol (HDL-C), low-density lipoprotein cholesterol (LDL-C), and 2hPG.

#### 2.2.5 Statistical analysis

Based on the distribution characteristics of continuous variables, means and standard deviations were calculated for normally distributed variables, whereas medians and interquartile ranges (IQRs) were reported for skewed continuous variables. For categorical variables, frequencies and percentages were calculated.

To evaluate the nonlinear relationship between the TyG index and cross-sectional glycemic status categories, this study applied generalized additive models (GAM) regression with smooth curve fitting to determine the optimal number of knots. Based on the optimal number of knots, restricted cubic spline (RCS) curves were plotted to test the statistical significance of nonlinearity and identify inflection point values, thereby informing clinical decision-making. Statistical rationale for the TyG inflection point. We specified the analytic logic as follows: we first identified the optimal number of knots from GAM smoothing, and then fitted RCS curves based on this optimal knot number to (i) test the statistical significance of the nonlinearity (edf and *P*-value) and (ii) mathematically identify the inflection point (the TyG value at which the slope of the dose-response curve changes most markedly, here TyG = 9.14). This inflection point is a data-driven estimate intended to inform risk stratification, and we now explicitly frame it as hypothesis-generating rather than a definitive clinical threshold.

Subgroup analyses were conducted using the fitted RCS model to assess heterogeneity, examine the consistency of the nonlinear association across different population subgroups, and enhance the generalizability of findings. GAM curves were generated for each cross-sectional glycemic status categories to enable stage-specific evaluation. Line graphs and box plots were constructed to facilitate path-specific analysis of these cross-sectional glycemic stages. To assess the reliability of the imputation procedure, we compared the main effect estimates from the imputed dataset with those from the complete-case analysis (n = 394). The results were consistent in terms of direction and statistical significance (**Supplementary Table S3**).

We employed two mediation methods to explore possible indirect associations through inflammatory indicators. First, the indirect mediating effect was quantified via regression path decomposition; second, the stability and statistical significance of the indirect effect were assessed using Quasi-Bayesian approximation with 1000 simulation iterations. If the mediating effect simultaneously meets the following two conditions, it is considered to be statistically significant: (1) In traditional regression analysis, both the coefficient *a* (effect of the TyG index on the inflammatory indicator, X→M) and the coefficient *b* (effect of the inflammatory indicator on stage transition risk after controlling for the TyG index, M→Y) were significant at the *P* < 0.05 level. (2) The 95% confidence interval of the indirect effect derived from the Quasi-Bayesian approximation excluded zero. In this model, coefficient *c’* represents the direct effect of the independent variable X on the dependent variable Y after adjusting for inflammatory indicators (X→Y). The indirect effect ACME is obtained by multiplying *a* and *b*.

Mediation analyses were conducted using the R software (version 4.5.3; R Foundation for Statistical Computing, https://www.r-project.org). Linear regression models were applied for path decomposition. The R package “mediation” was used to conduct mediation effect testing based on 1000 Quasi-Bayesian simulation iterations, with robust standard errors specified for all models. The standardized path coefficients were extracted using the “lavaan” package. Statistical significance was defined as a two-sided *P* < 0.05. To correct for multiple comparisons, the Benjamini-Hochberg (BH) method was applied to control the false discovery rate (FDR) (more details see **Supplementary Figure A2** in the supplementary). Because all variables were measured concurrently in this cross-sectional design, our mediation analysis is exploratory and cannot establish temporal ordering or causality. Therefore, the indirect estimates are interpreted as statistical indirect associations rather than causal mediation effects.

## 3 Results

### 3.1 Baseline characteristics of participants

The demographic and clinical baseline characteristics of the study participants are presented in **Table 1**. This table displays the distribution of variables across TyG index quartiles (Q_1_–Q_4_) and the corresponding *P*-values for interquartile differences. Age distribution exhibited an inverted U-shaped pattern. TyG index levels were highest among middle-aged participants (Q_2_–Q_3_) and lower among younger and older participants (Q_1_ and Q_4_). As TyG index values increased across quartiles, the proportion of males increased progressively, whereas the proportion of females decreased (*P* < 0.001), suggesting that insulin resistance may be more prevalent or pronounced in men than in women. With increasing TyG index quartile, FPG, 2hPG, and hemoglobin A1c (HbA1c) showed significant upward trends (all *P* < 0.001), indicating a strong association between the TyG index and severity of dysglycemia. The TyG index was significantly associated with the lipid profile: participants in the highest TyG index quartile (Q_4_) exhibited classic insulin resistance-associated dyslipidemia, elevated triglycerides, and reduced HDL-C. The association between the TyG index and systemic inflammation appears to be primarily mediated through the lipid metabolism pathway rather than exclusively via hematologic inflammatory markers. Finally, a significant dose-response relationship was observed between the TyG index quartile and T2DM prevalence (*P* < 0.001).

**Table 1:** Baseline characteristics of participants.

Variable	Quartiles of TyG index				*P-*value
	Q_1_ (n =280)	Q_2_ (n = 279)	Q_3_ (n = 279)	Q_4_ (n = 279)	
TyG	8.34 ± 0.27	8.93 ± 0.12	9.37 ± 0.15	10.18 ± 0.55	<0.001
Age	49.09 ± 16.98	52.57 ± 15.16	52.26 ± 14.63	47.76 ± 14.42	<0.001
Sex (%)					<0.001
Male	42.9%	47.7%	54.8%	63.8%	
Female	57.1%	52.3%	45.2%	36.2%	
FPG [mmol/L]	5.50 ± 1.06	6.36 ± 1.41	6.97 ± 1.90	8.80 ± 8.05	<0.001
2hPG [mmol/L]	9.42 ± 3.72	11.40 ± 4.47	13.07 ± 4.71	14.41 ± 5.94	<0.001
HbA1c	6.18 ± 1.04	6.53 ± 1.24	6.95 ± 1.66	7.58 ± 1.92	<0.001
TG [mmol/L]	1.01 ± 0.27	1.56 ± 0.34	2.25 ± 0.56	4.83 ± 3.23	<0.001
TC [mmol/L]	4.22 ± 1.10	4.31 ± 1.13	4.51 ± 1.10	5.13 ± 1.28	<0.001
HDL [mmol/L]	1.18 ± 0.31	1.10 ± 0.28	1.03 ± 0.27	0.91 ± 0.24	<0.001
LDL [mmol/L]	2.59 ± 0.87	2.70 ± 0.93	2.78 ± 0.92	2.89 ± 0.91	0.002
AISI	198.12 (108.72,340.07)	206.50 (115.29,370.42)	205.98 (115.11,374.17)	234.00 (131.28,375.92)	0.585
NLR	1.82 (1.42,2.93)	1.88 (1.47,3.03)	1.87 (1.54,3.06)	1.87 (1.54,2.80)	0.603
MLR	0.23 (0.16,0.32)	0.24 (0.16,0.32)	0.23 (0.16,0.31)	0.24 (0.18,0.33)	0.392
NMLR	2.03 (1.35,3.05)	2.12 (1.46,3.15)	2.11 (1.45,3.07)	2.13 (1.58,2.94)	0.639
SIRI	0.86 (0.45,1.40)	0.91 (0.46,1.46)	0.93 (0.56,1.43)	0.97 (0.57,1.50)	0.590
SII	440.74 (296.86,629.18)	446.69 (287.44,710.69)	421.97 (275.06,672.62)	465.10 (301.35,705.42)	0.411
LHR	1.68 (1.25,2.32)	1.84 (1.38,2.44)	2.06 (1.48,2.81)	2.36 (1.75,3.33)	0.111
LLR	5.01 (3.47,7.28)	5.30 (3.49,7.56)	5.57 (3.67,7.89)	5.86 (3.94,8.53)	0.336
MHR	0.45 ± 0.25	0.51 ± 0.30	0.55 ± 0.31	0.62 ± 0.31	<0.001
MLDLR	1.25 ± 0.62	1.39 ± 0.75	1.42 ± 0.75	1.51 ± 0.74	<0.001
NLDLR	10.45 ± 6.32	11.11 ± 6.62	12.32 ± 7.87	12.99 ± 7.75	<0.001
NHDLR	3.78 ± 2.75	4.11 ± 2.73	4.76 ± 3.45	5.36 ± 3.22	<0.001
T2DM					<0.001
No	92.9%	78.1%	64.5%	49.1%	
Yes	7.1%	21.9%	35.5%	50.9%	

TyG, triglyceride-glucose; FPG, fasting plasma glucose; 2hPG, 2-hour postprandial plasma glucose; HbA1c, hemoglobin A1c; TG, triglycerides; TC, total cholesterol; HDL, high-density lipoprotein cholesterol; LDL, low-density lipoprotein cholesterol; T2DM, type 2 diabetes mellitus; AISI = (Neutrophil count × Platelet count × Monocyte count)/Lymphocyte count; SIRI = (Neutrophil count × Monocyte count)/Lymphocyte count; NMLR = (Monocyte count + Neutrophil count)/Lymphocyte count; MLR = (Monocyte count/Lymphocyte count); NLR = (Neutrophil count/Lymphocyte count); SII = (Platelet count × Neutrophil count)/Lymphocyte count; LHR = (Lymphocytes/HDL); LLR = (Lymphocytes * LDL); MHR = (Monocytes/HDL); MLDLR = (Monocytes * LDL); NLDLR = (Neutrophils * LDL); NHDLR = (Neutrophils/HDL).

### 3.2 Nonlinear relationship between TyG index and T2DM

As shown in **Figure 1a**, the GAM smoothing curve demonstrates that the predicted probability of T2DM increases with the TyG index. After adjusting for age, sex, 2hPG, TC, HDL, and LDL, the association between the TyG index and T2DM risk remains statistically significant (edf = 3.27, *P* < 0.001), which indicates a nonlinear relationship. **Figure 1b** presents the RCS curve with four knots, which identifies an inflection point at TyG = 9.14. When the TyG index exceeds 9.14, the risk of T2DM (expressed as odds ratio) increases rapidly with rising TyG index values, and the overall nonlinear association is statistically significant (*P* < 0.001).

**Figure 1:**
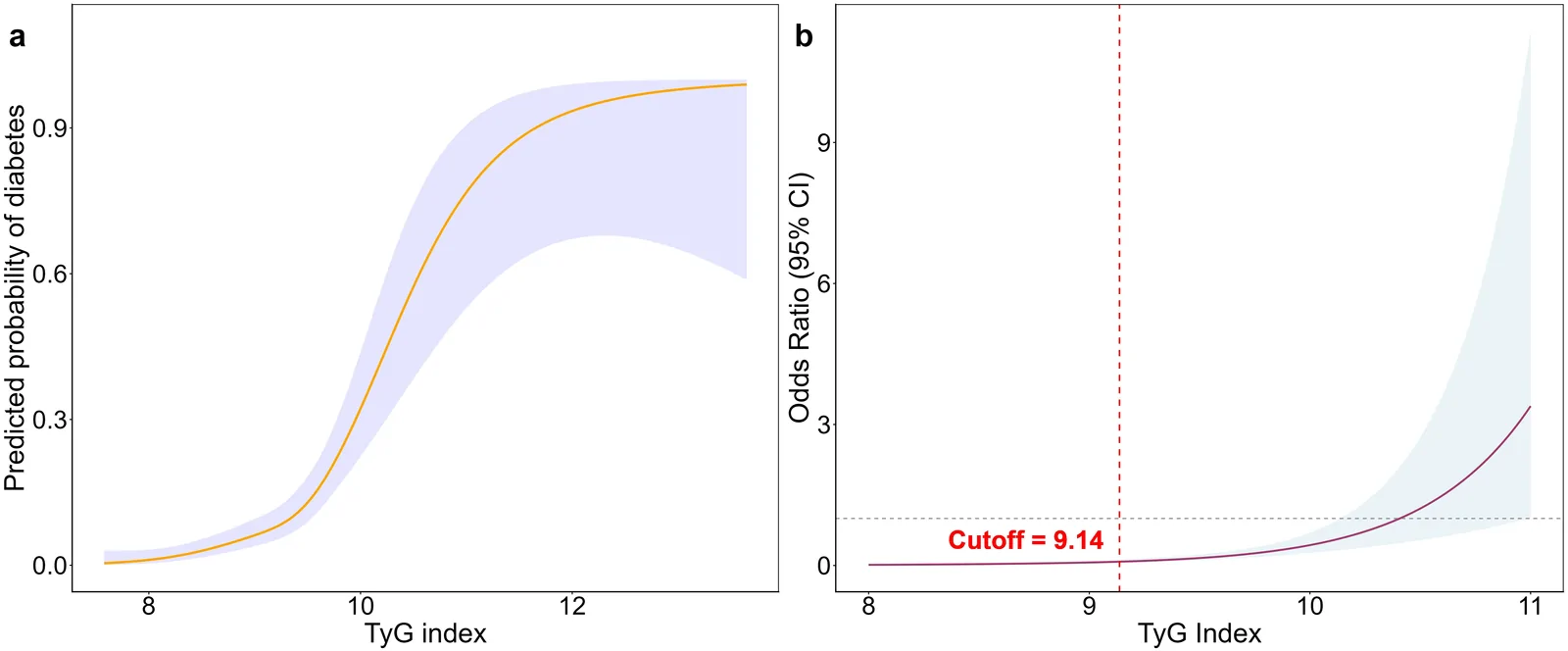
Nonlinear relationship between TyG index and T2DM. **(a)** GAM smoothing curve showing the predicted probability of T2DM across TyG index values. **(b)** Restricted cubic spline (RCS) curve with 4 knots, identifying an inflection point at TyG = 9.14. Models were adjusted for age, sex, 2hPG, TC, HDL, and LDL. GAM, generalized additive model; RCS, restricted cubic spline; T2DM, type 2 diabetes mellitus; TyG, triglyceride-glucose; 2hPG, 2-hour postprandial plasma glucose; TC, total cholesterol; HDL, high-density lipoprotein cholesterol; LDL, low-density lipoprotein cholesterol.

To explore the stability and heterogeneity of the association between the TyG index and T2DM, as given in **Figure 2**, subgroup analyses were performed by age, sex, 2hPG, TC, HDL, and LDL, respectively. We observed that the overall result presented was largely consistent. This indicates that the association is robust and not substantially influenced by subgroup-specific characteristics.

**Figure 2:**
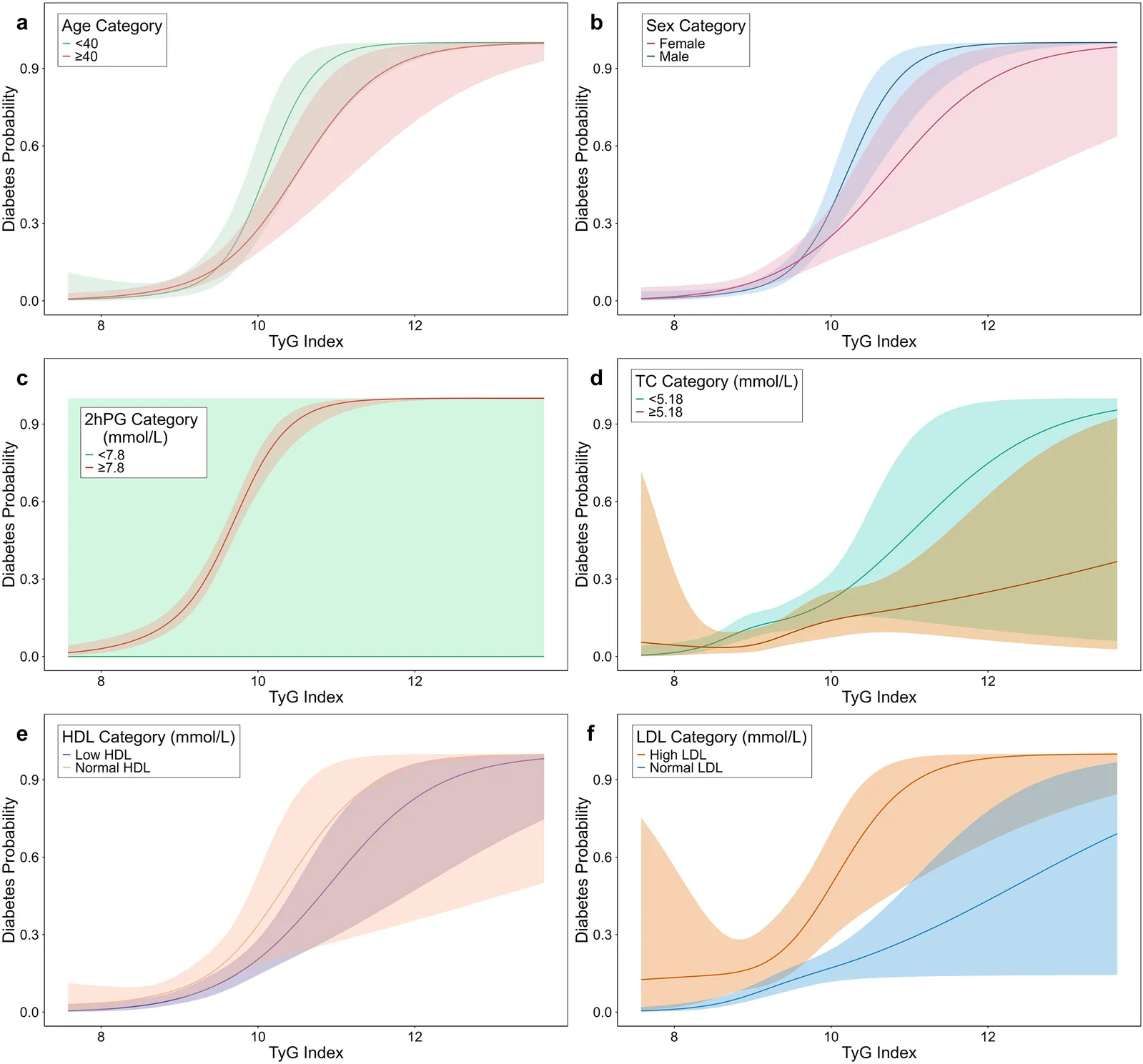
Subgroup analysis of the nonlinear relationship between TyG index and T2DM. **(a)** stratified by Age, **(b)** stratified by Sex, **(c)** stratified by 2hPG, **(d)** stratified by TC, **(e)** stratified by HDL, **(f)** stratified by LDL. TyG, triglyceride-glucose; T2DM, type 2 diabetes mellitus; 2hPG, 2-hour postprandial plasma glucose; TC, total cholesterol; HDL, high-density lipoprotein cholesterol; LDL, low-density lipoprotein cholesterol.

### 3.3 Nonlinear association between the TyG index and cross-sectional glycemic status categories

As shown in **Figure 1**, the results indicate a nonlinear association between the TyG index and cross-sectional glycemic status categories. Specifically, in the early stage of insulin resistance, the increase in diabetes risk is not statistically significant. When the TyG index exceeds a specific threshold, a significant association emerges. However, when the TyG index reaches very high levels, this association plateaus. To investigate whether this pattern is consistent with the role of insulin resistance across the glycemic spectrum and its association with dysglycemia, we performed stage-specific analyses across cross-sectional glycemic categories. The results are shown in **Figure 3**.

**Figure 3:**
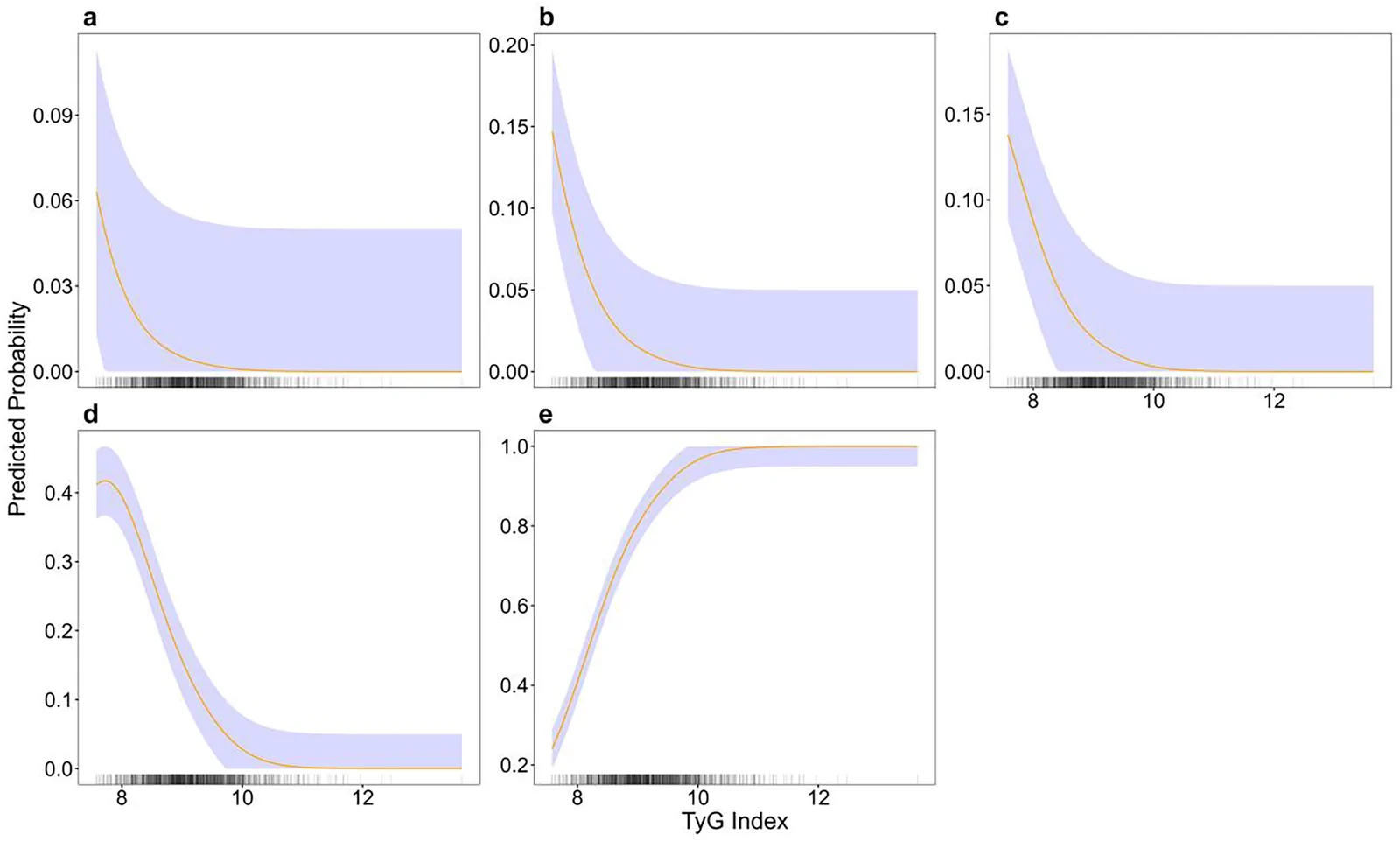
The GAM smooth curve of the TyG index and cross-sectional glycemic stages. **(a)** Normal Glucose Tolerance (n=178), **(b)** Impaired Fasting Glucose(n=60), **(c)** Impaired Glucose Tolerance(n=109), **(d)** Mixed Glucose Intolerance(n=126), **(e)** Type 2 Diabetes Mellitus(n=644). GAM, generalized additive model; TyG, triglyceride-glucose; T2DM, type 2 diabetes mellitus; NGT, normal glucose tolerance; IFG, impaired fasting glucose; IGT, impaired glucose tolerance.

The lower the TyG index, the higher the probability of normal glucose tolerance. Conversely, as the TyG index increases, the probability of maintaining normoglycemia may decrease significantly. With increasing TyG index, the probabilities of IFG and IGT also decline, which implies that the TyG index may be associated with a broader spectrum of metabolic abnormalities. However, the confidence intervals for these three stages are relatively wide, likely due to insufficient sample size. In the IFG+IGT stage, the curve initially rises slightly with increasing TyG index, then declines rapidly, and stabilizes when the TyG index reaches approximately 10. This suggests that, at low TyG index values, the probability of abnormal mixed glucose metabolism is slightly elevated, as the TyG index increases, this probability decreases markedly. The T2DM stage curve continues to rise with increasing TyG index and remains high in the upper TyG index range. These findings indicate that the TyG index can serve as an effective indicator for identifying individuals at high risk of diabetes. A high TyG index is associated with greater odds of the T2DM phenotype among those with prediabetic glucose abnormalities.

As shown in **Figure 4**, we also illustrate how the TyG index changes across cross-sectional glycemic stages:

**Figure 4:**
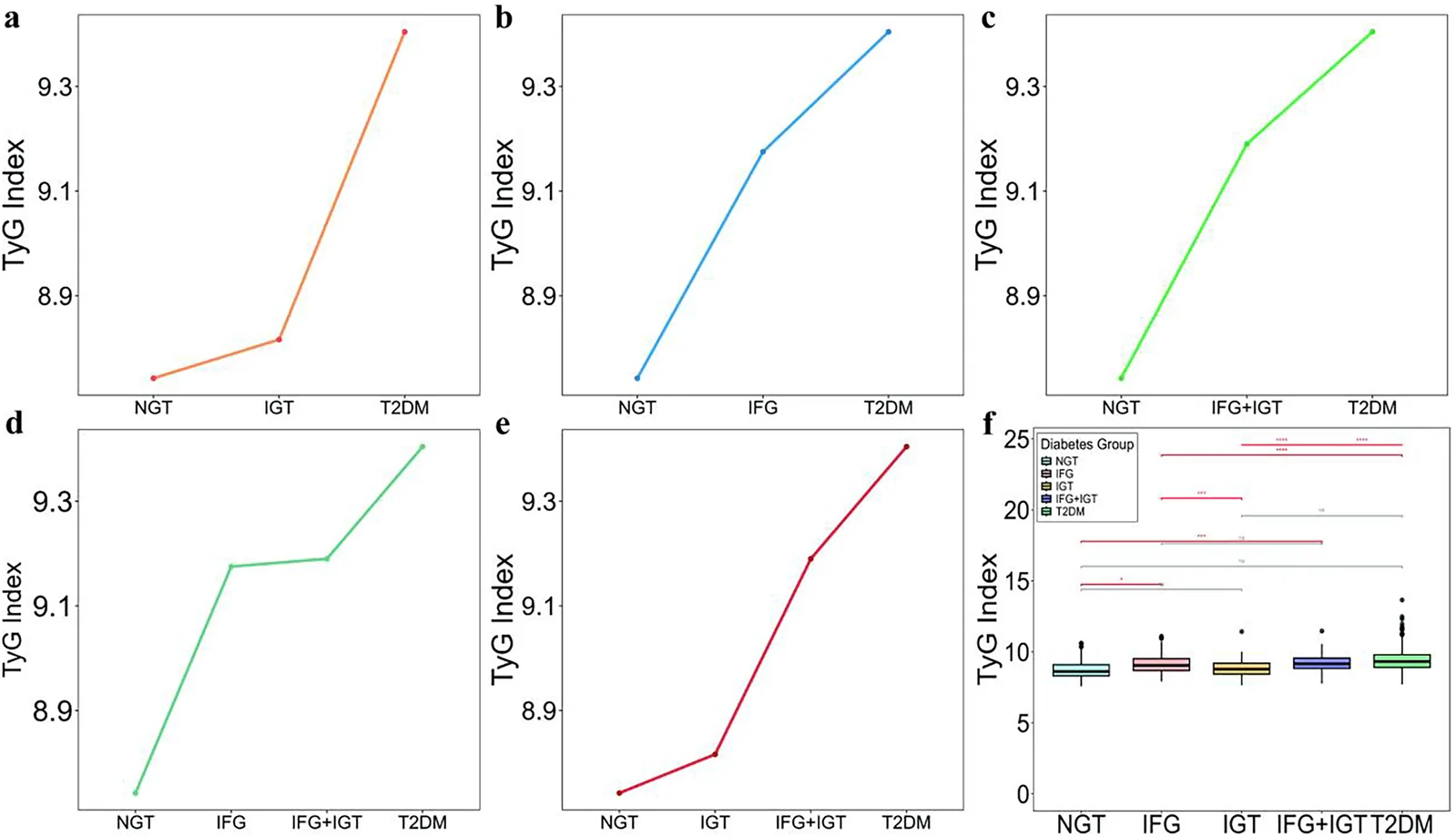
Trends in the TyG index across cross-sectional glycemic categories. **(a)** Pathway: NGT→IGT→T2DM, **(b)** Pathway: NGT→IFG→T2DM, **(c)** Pathway: NGT→IFG+IGT→T2DM, **(d)** Pathway: NGT→IFG→IFG+IGT→T2DM, **(e)** Pathway: NGT→IGT→IFG+IGT→T2DM, **(f)** Box plots of the TyG index across cross-sectional glycemic categories. TyG, triglyceride-glucose; T2DM, type 2 diabetes mellitus; NGT, normal glucose tolerance; IFG, impaired fasting glucose; IGT, impaired glucose tolerance.

Pathway 1: NGT→IGT→T2DM. Starting from the low value in the NGT stage, it slightly rises in the IGT stage, and reaches the most significant jump in the T2DM stage, reaching the peak. This indicates that during the conversion process from IGT to T2DM, the TyG index experiences the most significant increase.

Pathway 2: NGT→IFG→T2DM. This path indicates that when IFG progresses to T2DM, the TyG index shows a stable and continuous upward trend. Compared to path 1, its rising pace is more gradual, but the cumulative effect is significant.

Pathway 3: NGT→IFG+IGT→T2DM. This pathway is similar to Pathway 2, but the IFG+IGT stage is the critical point where the TyG index rises. Once this stage is entered, the TyG index is already at a relatively high level, and the risk of developing T2DM increases significantly.

Pathway 4: NGT→IFG→IFG+IGT→T2DM. This stage shows a gradual increase from the NGT stage to the IFG stage, becomes relatively stable from the IFG stage to the IFG+IGT stage, and then experiences explosive growth in the T2DM stage.

Pathway 5: NGT→IGT→IFG+IGT→T2DM. This trend gradually rises from NGT to IGT, accelerates from IGT to IFG+IGT, and reaches its peak in the T2DM stage.

As diabetes progresses from normal glucose tolerance through various prediabetes stages (IGT, IFG, and IFG+IGT) to T2DM, the TyG index shows a significant and robust upward trend (*P* = 0.000), peaking in the T2DM stage. In **Figure 4f**, the Kruskal–Wallis test indicates a significant difference in the distribution of the TyG index across cross-sectional glycemic stages (*χ²*(4)=158.66, *P* < 0.001), and the effect size is moderate (ϵ² ≈ 0.14). The TyG index shows a significant gradient change across cross-sectional glycemic stages. The TyG index in the T2DM group was significantly higher than that in all other groups, and may serve as a key metabolic characteristic for distinguishing T2DM from prediabetic conditions.

### 3.4 Exploratory analysis of inflammatory indicators for indirect associations

To explore potential inflammatory factors associated with glycemic phenotypes across cross-sectional glycemic stages, this study constructed 12 inflammatory indicators as possible mediating variables. We employed 2hPG as continuous outcome variable to quantitatively assess the indirect effect of these inflammatory indicators on the relationship between the TyG index and blood glucose levels. All models were adjusted for the covariates of age and sex.

Quantitatively, for 2hPG, each one-unit increase in the TyG index was associated with a significant indirect increment of 0.070 units (95% CI: 0.019–0.130, corrected *P* = 0.048) through MLDLR, and 0.060 units (95% CI: 0.016–0.124, corrected *P* = 0.048) via NLDLR. The results are shown in **Figure 5**. These positive indirect effects may suggest that an elevated TyG index is associated with increased levels of these inflammatory factors, which may contribute to elevated 2hPG levels. The indirect effects of the other inflammatory factors were not statistically significant after multiple test corrections (all *P* > 0.05). (For more details, please refer to **Supplementary Tables S1**, **S2** in the **Supplementary Material**).

**Figure 5:**
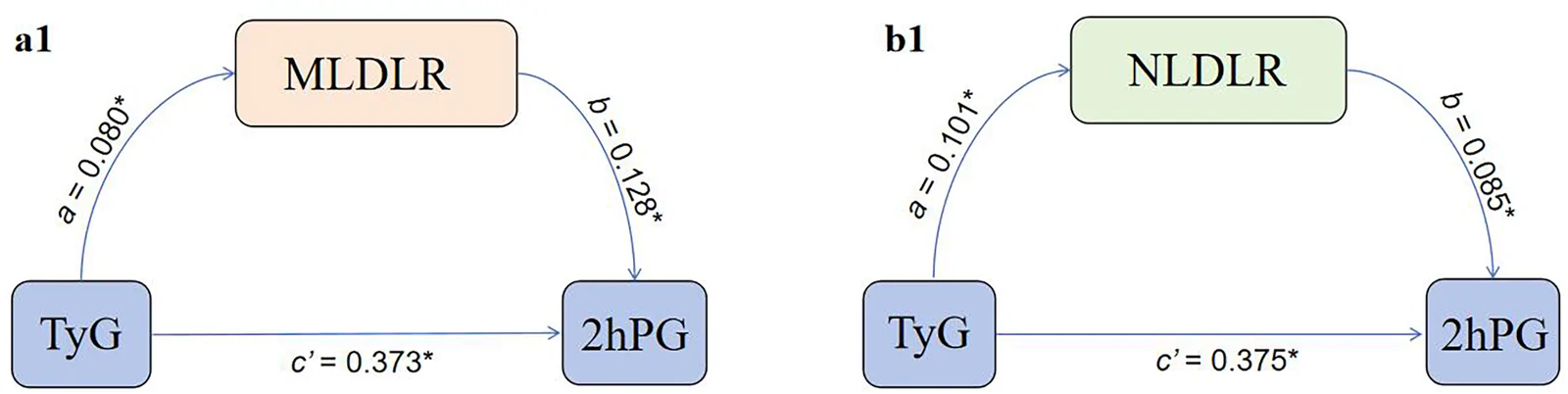
Path coefficient diagrams for the possible indirect effects of inflammatory indicators between the TyG index and 2hPG. (a1) Path coefficient diagram with MLDLR as the mediating variable, (b1) Path coefficient diagram with NLDLR as the mediating variable. TyG, triglyceride-glucose; 2hPG, 2-hour postprandial plasma glucose; MLDLR = (Monocytes * LDL); NLDLR = (Neutrophils * LDL). *P < 0.05.

As a sensitivity analysis, we repeated the exploratory mediation analyses using the complete-case subset (n=394). In this subset, none of the 12 inflammatory indicators showed statistically significant mediating effects. This discrepancy is likely attributable to reduced statistical power in the smaller sample (n=394) compared with the imputed dataset (n=1,117). The complete-case results are provided in **Supplementary Table S4**.

## 4 Discussion

The progression of T2DM is not a uniform process; it may follow distinct pathophysiological pathways that evolve across different disease stages. The etiology of chronic non-communicable diseases is intricate and multifactorial, with dynamic interactions among multiple factors (24). This nonlinear pattern suggests that simple linear models cannot adequately describe the association and underscores the importance of considering heterogeneity across cross-sectional glycemic categories. In this study, we focused on identifying whether the TyG index exhibits stage-specific nonlinear patterns across different hypothesized pathways of glycemic dysregulation. These analyses confirmed a significant nonlinear association: across successive cross-sectional glycemic stages, the TyG index shows a markedly upward trend that varies in slope and magnitude depending on the glycemic pathway. We further examined whether inflammatory indicators are involved in the observed nonlinear associations via indirect statistical links. Exploratory analyses of indirect associations suggested that inflammatory indicators may partly explain the association between the TyG index and cross-sectional glycemic categories. These indirect associations appeared to be category-specific and indicator-dependent.

Although the hyperinsulinemic-euglycemic clamp (HEC) technique is considered the gold standard for measuring insulin resistance, it is technically demanding and costly, making it unsuitable for large-scale screening (25). In contrast, the TyG index, as a surrogate marker of insulin resistance, is a simple composite marker calculated from fasting plasma glucose and fasting triglycerides, which reliably reflects insulin resistance (7). Importantly, it does not require insulin measurement and relies solely on routine biochemical parameters. It is simple to calculate, cost-effective, and well suited for population-level screening, enabling early identification of individuals at high risk for T2DM and facilitating timely intervention. Given that insulin resistance is a primary pathophysiological driver of T2DM, using the TyG index as the independent variable is methodologically sound.

RCS analysis estimated a data-driven inflection point for the TyG index at approximately 9.14. Above this threshold, the probability of being in a more impaired glycemic category increased sharply in a nonlinear manner. This threshold is derived from routine clinical laboratory measurements and is associated with cross-sectional glycemic categories. It may aid in risk stratification among individuals with prediabetes and, after prospective validation, could serve as a practical reference for primary care. Clinicians in primary care settings should deliver early lifestyle interventions and regular 2hPG monitoring for patients with a TyG index above 9.14 to screen T2DM high-risk individuals and improve the clinical applicability of the TyG index. The findings align with those of multiple cohort and cross-sectional studies. For example, Ola Taha et al. reported in a prospective study that the TyG index was strongly positively correlated with HbA1c, fasting plasma glucose, HOMA-IR, and 2-hour postprandial glucose, and demonstrated robust predictive ability for type 2 diabetes, impaired glucose tolerance, impaired fasting glucose, and insulin resistance (26).

It is noteworthy that this study observed differentiated increases in the TyG index across distinct hypothesized pathways of glycemic dysregulation. In the NGT→IFG→T2DM pathway, the TyG index is strongly associated with the long-term cumulative risk of impaired fasting glucose, suggesting that the transition from normoglycemia to T2DM may be more protracted than in the NGT→IGT→T2DM pathway. In the latter pathway, the TyG index exhibits a pronounced increase at the IGT stage and indicates that it is an early-warning value for individuals with impaired glucose tolerance. The finding is consistent with the view expressed in reference that the TyG index is a useful biomarker for recognizing glucose metabolism disorders in apparently healthy children and adolescents (27) while also supporting the notion that the TyG index is sensitive to early glucose metabolism abnormalities (28). Furthermore, the TyG index is markedly elevated in the IFG+IGT group, suggesting that coexisting glycolipid metabolic disturbances exert a synergistic effect on lipid metabolism. These results provide evidence to support clinical risk stratification of high-risk populations.

The TyG index shows robust predictive performance and consistent utility across all prediabetes stages, while also displaying differential sensitivity to various glucose metabolism disorders. The magnitude of its increase may reflect differences in insulin resistance severity or nature under different pathological conditions. Notably, the IFG+IGT stage marks the steepest rise in the TyG index, representing a critical window for intervention. In **Figure 4f**, certain pairwise differences between stages were not statistically significant, suggesting either a gradual TyG increase during prediabetes with a sharp rise upon T2DM onset, or insufficient statistical power due to sample size. These findings suggest the potential utility of the TyG index as a continuous marker for differentiating among cross-sectional glycemic categories.

A large body of prior evidence confirmed that IL-6 (29), IL-1β (30), and TNF-α (31) were associated with glycemic dysregulation in T2DM. Due to limited sample availability for measuring these specific inflammatory markers, this study constructed a novel composite inflammatory indicator. These constituent variables are easily accessible and routinely measured in clinical practice.

Our exploratory analysis of indirect associations suggests that MLDLR and NLDLR show significant positive indirect associations in the relationship between the TyG index and 2hPG, with the indirect association for MLDLR being slightly larger than that for NLDLR. We speculate that an elevated TyG index may reflect systemic glucose–lipid metabolic dysregulation and aggravated insulin resistance, which could be linked to neutrophil proliferation (32), attenuated anti-inflammatory function of HDL (12), and upregulation of MLDLR and NLDLR. These alterations might in turn be associated with insulin resistance in skeletal muscle (33) and impaired postprandial pancreatic function (34), potentially contributing to elevated 2-hour postprandial glucose levels. However, this proposed pathway is speculative and requires experimental validation. Moreover, given the cross-sectional nature of the present data, we cannot establish the temporal ordering among the exposure, putative mediators, and outcomes. Therefore, the observed indirect associations should be interpreted as exploratory, hypothesis-generating findings rather than as definitive evidence of causal mediation. Reverse causation and unmeasured common-cause confounding cannot be fully excluded. Large prospective longitudinal studies with temporally separated measurements are needed to validate the proposed mechanistic pathway.

While the TyG index and 2hPG both reflect insulin resistance and glucose dysmetabolism, they capture distinct metabolic phenotypes. The TyG index integrates fasting triglycerides and fasting glucose, mainly reflecting fasting-state insulin resistance driven by glucolipotoxicity. In comparison, 2hPG focuses on postprandial glucose homeostasis, reflecting dynamic glucose clearance capacity and pancreatic β-cell compensatory secretory function after a glucose load. Postprandial glycemic disturbance is an early and pivotal abnormality across the glycemic spectrum of dysglycemia and cannot be fully represented by fasting metabolic phenotypes alone. Therefore, exploring the indirect association between the TyG index and 2hPG involving inflammatory indicators helps elucidate the potential mechanism by which chronic basal glycolipid metabolic dysfunction exacerbates postprandial glycemic dysregulation via inflammation. This provides novel epidemiological evidence for understanding differences in glycemic status across cross-sectional glycemic categories, from normal glucose to T2DM.

Nevertheless, our exploratory analysis of indirect associations identified MLDLR and NLDLR as factors showing potential indirect links in the association between the TyG index and 2hPG. As composite inflammatory markers that are readily derivable from routine complete blood counts and lipid panels, these two indicators hold practical value for risk stratification in glucose metabolism disorders and may inform adjunctive anti-inflammatory strategies in individuals with elevated TyG index. Given their clinical accessibility, future prospective intervention studies are warranted to validate whether targeted modulation of MLDLR and NLDLR confers tangible benefits in ameliorating dysglycemic phenotypes, which is a crucial step before these markers can be translated into routine practice.

To further assess the robustness of the results and generalizability, we conducted subgroup analyses stratified by age, sex, 2hPG, HDL-C, TC, and LDL-C. Using 40 years as the cutoff (35), the TyG index showed higher predictive accuracy for incident diabetes in the < 40-year group, indicating better early-warning sensitivity in younger individuals. Consistent with sexr-specific differences in fat distribution (36, 37), men exhibited a higher diabetes prevalence than women at equivalent TyG index levels. For 2hPG glucose, the TyG index’s peak risk association was attenuated when glucose < 7.8 mmol/L but intensified when ≥ 7.8 mmol/L, suggesting that baseline glucose status modulates its predictive performance. In TC-stratified analysis, individuals with normal TC (< 5.18 mmol/L) had a significantly higher diabetes risk at equivalent TyG index levels than those with elevated TC (≥5.18 mmol/L). Similarly, the risk curve for the normal HDL-C group lay above that of the low HDL-C group. These findings indicate that the TyG index has greater predictive value and sensitivity to insulin resistance shifts in individuals without underlying dyslipidemia, aligning with Sun et al. (2025) (38). In contrast, among those with pre-existing dyslipidemia, diabetes risk is driven jointly by lipid abnormalities and insulin resistance (10), thereby attenuating the TyG index’s predictive signal. For LDL-C, individuals with high LDL-C showed significantly elevated diabetes risk at matched TyG index levels relative to those with normal LDL-C. This is consistent with current literature: elevated LDL-C promotes hepatic and peripheral lipid deposition, impairing insulin signaling and exacerbating insulin resistance (39), while lipid overload activates systemic low-grade inflammation contributing to pancreatic β-cell dysfunction (40). These two processes may be related to the insulin resistance reflected by the TyG index and were associated with more impaired glycemic categories across cross-sectional glycemic status categories, from normal glucose to T2DM.

This study has some limitations. First, regarding study design, it is a retrospective analysis of cross-sectional data that can only identify associations, rather than causal relationships, between the TyG index and cross-sectional glycemic categories, from normal glucose to T2DM. Additionally, the sample size is relatively small, leading to wide confidence intervals and potentially imbalanced subgroup distributions; thus, the robustness of the findings requires further validation. Second, with respect to indicator accuracy, fasting plasma glucose is subject to multiple physiological and preanalytical confounders, which may attenuate the TyG index’s predictive performance. Despite HbA1c being a stable and integrated marker of glycemic exposure, its high rate of missing data precluded its inclusion in this study. Future research should investigate composite indices incorporating HbA1c for T2DM risk prediction and carefully assess their agreement with the hyperinsulinemic-euglycemic clamp technique as the diagnostic reference standard. Third, we acknowledge the lack of complete baseline medication records. While the use of first-visit laboratory values minimizes the influence of chronic pretreatment, it does not entirely exclude prior therapies prescribed outside our hospital, leaving residual confounding as a possible limitation. We therefore did not adjust for medication status in the mediation models, which might affect the robustness of our estimates. Future prospective studies incorporating comprehensive drug histories are warranted, and additional analytical strategies, such as sensitivity analyses or instrumental variable methods, could help further disentangle the confounding effects of these medications on the mediating pathways. Fourthly, while the imputed dataset identified MLDLR and NLDLR as significant mediators, this finding was not replicated in the complete-case analysis (n=394), likely due to the substantially smaller sample size and consequent loss of statistical power. This discrepancy highlights the need for cautious interpretation and further validation in larger prospective cohorts with complete data collection. Fifth, because exposure, mediators and outcomes were measured concurrently in this cross-sectional retrospective design, our mediation analysis cannot verify temporal sequence, limiting causal interpretation. Observed indirect effects are exploratory statistical associations rather than proven causal pathways. In future studies, we intend to develop disease progression models using routinely available clinical data, should conditions permit. Validation of these findings would help streamline risk assessment and facilitate precision intervention in diabetes prevention.

## 5 Conclusions

This study systematically examined the association between the TyG index and diabetes across distinct prediabetes stages and clarified the mediating role of inflammatory markers. GAM, RCS analyses, and subgroup analyses consistently revealed a nonlinear association between the TyG index and diabetes risk. In this cross-sectional analysis, the TyG index was nonlinearly associated with glycemic status categories, with higher TyG values observed in categories reflecting greater glucose impairment and a data-driven inflection point at approximately 9.14. This inflection point should be regarded as hypothesis-generating pending prospective validation. The mediation analysis suggested exploratory indirect associations through MLDLR and NLDLR in the relationship between the TyG index and 2hPG. These findings are consistent with, but do not provide proof of, an inflammation-related pathway. Given the cross-sectional design and the fact that the significant indirect associations observed in the primary multiple-imputation analysis were not reproduced in the complete-case analysis, these results require confirmation in large prospective cohorts before application in clinical or primary-care settings.

## Data Availability

The data on which this research is based are currently not made public. These data are sourced from the electronic medical record system of the Ningxia General Hospital in China. Due to institutional and ethical restrictions, they involve patient privacy and confidentiality requirements. As the data contain potentially identifiable and sensitive clinical information, access requires approval by the hospital’s Institutional Review Board (IRB) and compliance with data governance policies. With the necessary permission from the hospital’s ethics committee and the signing of relevant data usage agreements, the authors can respond to reasonable requests to obtain this data. Requests to access these datasets should be directed to YZ, zhaoyu@nxmu.edu.cn.

## References

[1] Elsayed NA Aleppo G Aroda VR Bannuru RR Brown FM Bruemmer D . Classification and diagnosis of diabetes: Standards of care in diabetes-2023. Diabetes Care. (2023) 46:S19–40. doi: 10.2337/dc23-S002 PMC9810477 36507649

[2] GBD 2021 Diabetes Collaborators . Global, regional, and national burden of diabetes from 1990 to 2021, with projections of prevalence to 2050: a systematic analysis for the Global Burden of Disease Study 2021. Lancet. (2023) 402:203–34. doi: 10.1016/S0140-6736(25)01635-6 PMC10364581 37356446

[3] Sadafi S Azizi A Najafi F Pasdar Y . Lipid accumulation product and type 2 diabetes risk: a population-based study. BMC Endocr Disord. (2024) 24:147. doi: 10.1186/s12902-024-01682-6 39134995 PMC11318136

[4] Gong Q Zhang P Wang J Ma J An Y Chen Y . Morbidity and mortality after lifestyle intervention for people with impaired glucose tolerance: 30-year results of the Da Qing Diabetes Prevention Outcome Study. Lancet Diabetes Endocrinol. (2019) 7:452–61. doi: 10.1016/s2213-8587(19)30093-2 31036503 PMC8172050

[5] Knowler WC Barrett-Connor E Fowler SE Hamman RF Lachin JM Walker EA . Reduction in the incidence of type 2 diabetes with lifestyle intervention or metformin. N Engl J Med. (2002) 346:393–403. doi: 10.1056/NEJMoa012512 PMC1370926 11832527

[6] Choo YN Ravi RN Subramaniyan V . Insulin resistance induced by obesity: Mechanisms, metabolic implications and therapeutic approaches. Mol Biol Rep. (2026) 53:357. doi: 10.1007/s11033-026-11509-3 41636917 PMC12872655

[7] Simental-Mendía LE Rodríguez-Morán M Guerrero-Romero F . The product of fasting glucose and triglycerides as surrogate for identifying insulin resistance in apparently healthy subjects. Metab Syndr Relat Disord. (2008) 6:299–304. doi: 10.1089/met.2008.0034 19067533

[8] Low S Khoo KCJ Irwan B Sum CF Subramaniam T Lim SC . The role of triglyceride glucose index in development of Type 2 diabetes mellitus. Diabetes Res Clin Pract. (2018) 143:43–9. doi: 10.1016/j.diabres.2018.06.006 29936253

[9] Mirabelli M Misiti R Sicilia L Brunetti FS Chiefari E Brunetti A . Hypoxia in human obesity: New insights from inflammation towards insulin resistance-a narrative review. Int J Mol Sci. (2024) 25:9802. doi: 10.3390/ijms25189802 39337290 PMC11432683

[10] Kahn SE Hull RL Utzschneider KM . Mechanisms linking obesity to insulin resistance and type 2 diabetes. Nature. (2006) 444:840–6. doi: 10.1038/nature05482 17167471

[11] Guariguata L Whiting DR Hambleton I Beagley J Linnenkamp U Shaw JE . Global estimates of diabetes prevalence for 2013 and projections for 2035. Diabetes Res Clin Pract. (2014) 103:137–49. doi: 10.1016/j.diabres.2013.11.002 24630390

[12] Adams-Huet B Jialal I . An increasing triglyceride-glucose index is associated with a pro-inflammatory and pro-oxidant phenotype. J Clin Med. (2024) 13:3941. doi: 10.3390/jcm13133941 38999506 PMC11242814

[13] Duan Y Zhang R Zhang Y Ma Y Li M Zhang W . Relationship between TYG mediated pregnancy diabetes history and SII in American women: a retrospective cohort study of NHANES 2011-2018. BMC Endocr Disord. (2025) 25:208. doi: 10.1186/s12902-025-02042-8 40983922 PMC12455841

[14] Wang Y Wang X Zeng L . Lipid accumulation product as a predictor of prediabetes and diabetes: Insights from NHANES data (1999-2018). J Diabetes Res. (2024) 2024:2874122. doi: 10.1155/2024/2874122 39559713 PMC11573446

[15] Xiu J Lin X Chen Q Yu P Lu J Yang Y . The aggregate index of systemic inflammation (AISI): a novel predictor for hypertension. Front Cardiovasc Med. (2023) 10:1163900. doi: 10.3389/fcvm.2023.1163900 37265570 PMC10229810

[16] Hu B Yang XR Xu Y Sun YF Sun C Guo W . Systemic immune-inflammation index predicts prognosis of patients after curative resection for hepatocellular carcinoma. Clin Cancer Res. (2014) 20:6212–22. doi: 10.1158/1078-0432.ccr-14-0442 25271081

[17] Li HL Dong LP Wei HL Li H Tian M Dong JW . Elevated neutrophil-monocyte-to-lymphocyte ratio increases risk of adverse outcomes in patients with chronic kidney disease and type 2 diabetes. Eur J Med Res. (2025) 30:436. doi: 10.1186/s40001-025-02671-8 40450343 PMC12125722

[18] Lin H Dong X Yin Y Gao Q Peng S Zhao Z . Exploring the influencing factors of abdominal aortic calcification events in chronic kidney disease (CKD) and non-CKD patients based on interpretable machine learning methods. Int Urol Nephrol. (2025) 57:3787–98. doi: 10.1007/s11255-025-04564-5 40348897

[19] Li D Fan Y He B Tang Y Deng G Guo R . The prognostic value of neutrophil-to-lymphocyte ratio and lymphocyte-to-monocyte ratio in patients with hepatocellular carcinoma receiving HAIC-based conversion hepatectomy: a dual-center retrospective cohort study. J Inflammation Res. (2025) 18:8675–88. doi: 10.2147/jir.s523194 40620605 PMC12229162

[20] Yu X Zheng H Liu M Wu L Tian S Wu W . Association of systemic immune-inflammation index with all-cause and cardiovascular mortality among adults with depression: evidence from NHANES 2005-2018. BMC Psychiatry. (2025) 25:25. doi: 10.1186/s12888-024-06463-y 39780080 PMC11707943

[21] Yuan S Pu T Wang Z Li L Gao P Zhang L . Correlation between neutrophil/lymphocyte ratio combined with low-density lipoprotein cholesterol/high-density lipoprotein cholesterol ratio and severity of coronary artery disease in patients with acute coronary syndrome. Zhonghua Wei Zhong Bing Ji Jiu Yi Xue. (2022) 34:274–9. doi: 10.3760/cma.j.cn121430-20211008-01441 35574745

[22] Sun M Zhao D Zhang Y Zhai Y Ye M Wang X . Prognostic utility of monocyte to high-density lipoprotein ratio in patients with acute coronary syndrome: a meta-analysis. Am J Med Sci. (2020) 359:281–6. doi: 10.1016/j.amjms.2020.01.018 32245567

[23] Lim HA Kang JK Kim HW Song H Lim JY . The neutrophil-to-lymphocyte ratio as a predictor of postoperative outcomes in patients undergoing coronary artery bypass grafting. J Chest Surg. (2023) 56:99–107. doi: 10.5090/jcs.22.082 36792946 PMC10008357

[24] Chen D Ma Y Xiao H Yan Z . Development trends of etiological research contents and methods of noncommunicable diseases. Health Care Sci. (2023) 2:352–7. doi: 10.1002/hcs2.69 38938587 PMC11080801

[25] Ramdas Nayak VK Satheesh P Shenoy MT Kalra S . Triglyceride glucose (TyG) index: a surrogate biomarker of insulin resistance. J Pak Med Assoc. (2022) 72:986–93. doi: 10.47391/JPMA.22-63 35713073

[26] Taha O Abdallah L Erfan A Elsharaby R El Amrousy D . The triglyceride glucose index as a biomarker for different glucose disorders in overweight and obese children and adolescents. Eur J Pediatr. (2025) 184:820. doi: 10.1007/s00431-025-06632-5 41345805 PMC12678614

[27] Simental-Mendía LE Gamboa-Gómez CI Aradillas-García C Rodríguez-Morán M Guerrero-Romero F . The triglyceride and glucose index is a useful biomarker to recognize glucose disorders in apparently healthy children and adolescents. Eur J Pediatr. (2020) 179:953–8. doi: 10.1007/s00431-020-03570-2 32016604

[28] Chen Z Wen J . Elevated triglyceride-glucose (TyG) index predicts impaired islet β-cell function: a hospital-based cross-sectional study. Front Endocrinol (Lausanne). (2022) 13:973655. doi: 10.3389/fendo.2022.973655 36246870 PMC9563389

[29] Kristiansen OP Mandrup-Poulsen T . Interleukin-6 and diabetes: the good, the bad, or the indifferent? Diabetes. (2005) 54:S114–24. doi: 10.2337/diabetes.54.suppl_2.s114 16306329

[30] Esser N Legrand-Poels S Piette J Scheen AJ Paquot N . Inflammation as a link between obesity, metabolic syndrome and type 2 diabetes. Diabetes Res Clin Pract. (2014) 105:141–50. doi: 10.1016/j.diabres.2014.04.006 24798950

[31] Hotamisligil GS Shargill NS Spiegelman BM . Adipose expression of tumor necrosis factor-alpha: direct role in obesity-linked insulin resistance. Science. (1993) 259:87–91. doi: 10.1126/science.7678183 7678183

[32] Feng YM Zhao D Zhang N Yu CG Zhang Q Thijs L . Insulin resistance in relation to lipids and inflammation in type-2 diabetic patients and non-diabetic people. PloS One. (2016) 11:e0153171. doi: 10.1371/journal.pone.0153171 27073920 PMC4830613

[33] Pedrini MT Kranebitter M Niederwanger A Kaser S Engl J Debbage P . Human triglyceride-rich lipoproteins impair glucose metabolism and insulin signalling in L6 skeletal muscle cells independently of non-esterified fatty acid levels. Diabetologia. (2005) 48:756–66. doi: 10.1007/s00125-005-1684-8 15747109

[34] Tu P Bhasin S Hruz PW Herbst KL Castellani LW Hua N . Genetic disruption of myostatin reduces the development of proatherogenic dyslipidemia and atherogenic lesions in Ldlr null mice. Diabetes. (2009) 58:1739–48. doi: 10.2337/db09-0349 19509018 PMC2712781

[35] Xu W Mu Y Zhao J Zhu D Ji Q Zhou Z . Efficacy and safety of metformin and sitagliptin based triple antihyperglycemic therapy (STRATEGY): a multicenter, randomized, controlled, non-inferiority clinical trial. Sci China Life Sci. (2017) 60:225–38. doi: 10.1007/s11427-016-0409-7 28271251

[36] Karastergiou K Smith SR Greenberg AS Fried SK . Sex differences in human adipose tissues - the biology of pear shape. Biol Sex Differ. (2012) 3:13. doi: 10.1186/2042-6410-3-13 22651247 PMC3411490

[37] Tramunt B Smati S Grandgeorge N Lenfant F Arnal JF Montagner A . Sex differences in metabolic regulation and diabetes susceptibility. Diabetologia. (2020) 63:453–61. doi: 10.1007/s00125-019-05040-3 PMC6997275 31754750

[38] Sun Y Ji H Sun W An X Lian F . Triglyceride glucose (TyG) index: a promising biomarker for diagnosis and treatment of different diseases. Eur J Intern Med. (2025) 131:3–14. doi: 10.1016/j.ejim.2024.08.026 39510865

[39] Jahdkaran M Sistanizad M . From lipids to glucose: Investigating the role of dyslipidemia in the risk of insulin resistance. J Steroid Biochem Mol Biol. (2025) 250:106744. doi: 10.1016/j.jsbmb.2025.106744 40158704

[40] Benito-Vicente A Jebari-Benslaiman S Galicia-Garcia U Larrea-Sebal A Uribe KB Martin C . Molecular mechanisms of lipotoxicity-induced pancreatic β-cell dysfunction. Int Rev Cell Mol Biol. (2021) 359:357–402. doi: 10.1016/bs.ircmb.2021.02.013 33832653

