# Supplementary material for: Association between triglyceride-glucose index and stages of T2DM: potential indirect effects of inflammatory indicators
Source: Front Endocrinol (Lausanne). 2026 Oct 2;17:1931822. doi: 10.3389/fendo.2026.1931822 (PMC13672205; doi:10.3389/fendo.2026.1931822)
Supplement: Supplementary file 1 [file DataSheet1.pdf]

## Supplementary

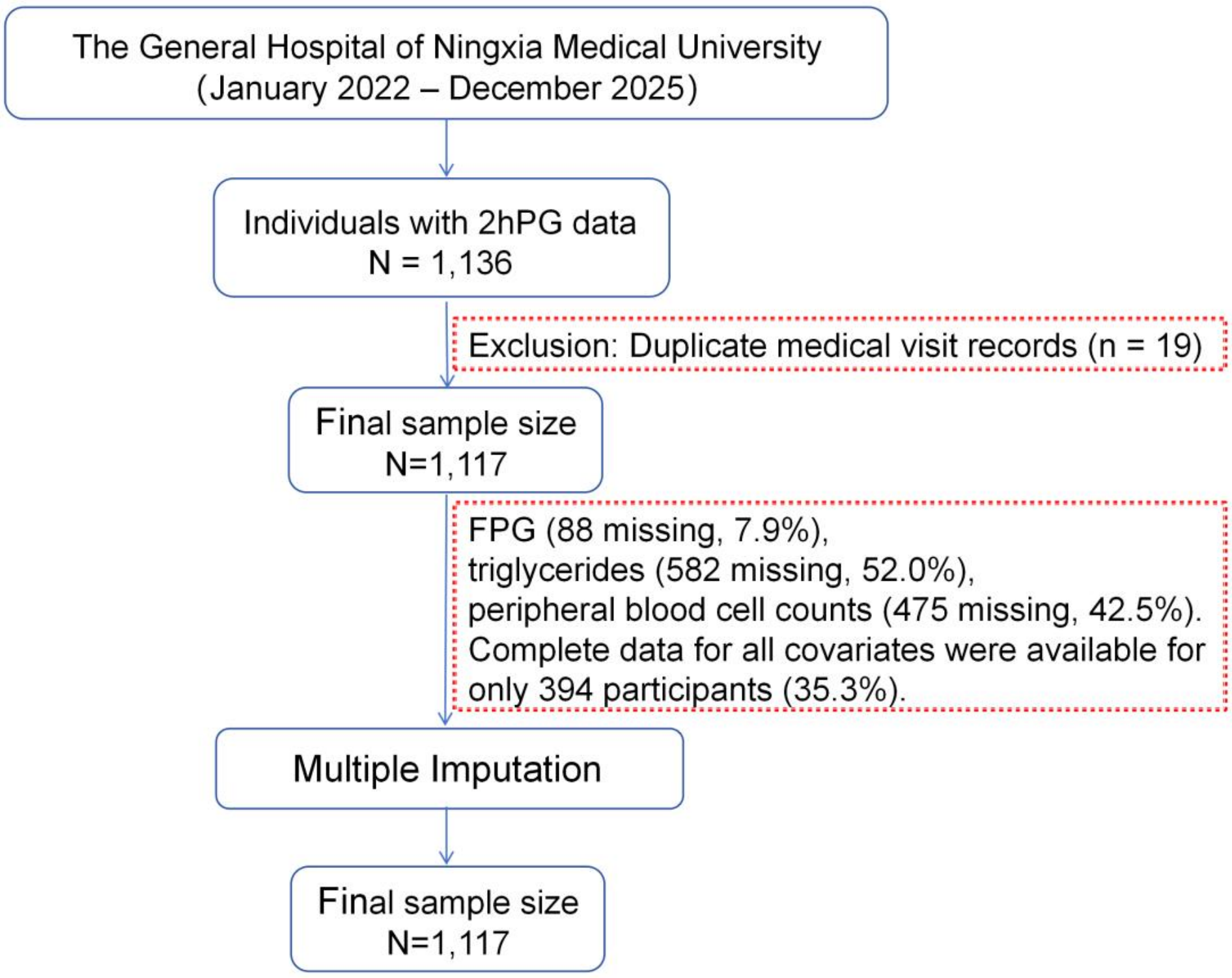


Figure A1. Flowchart of the Study Population Inclusion

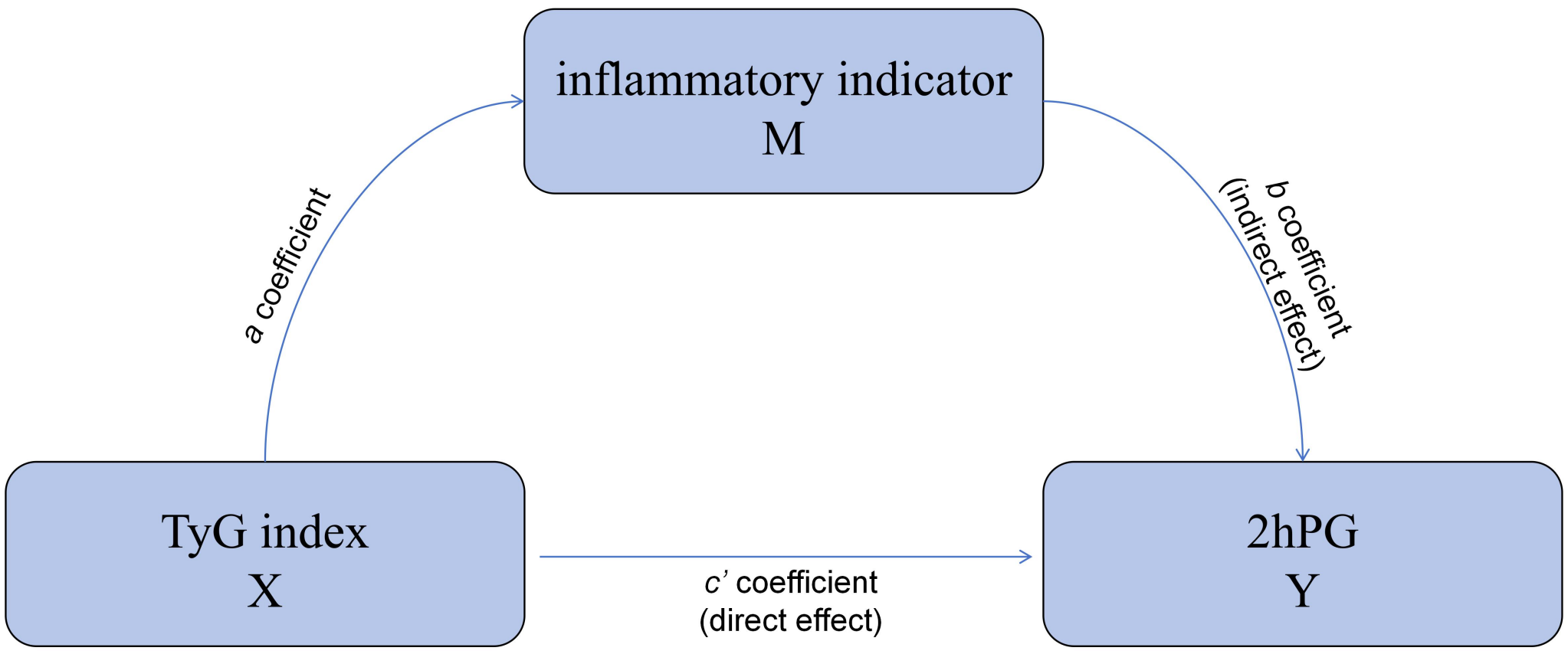


Figure A2. Path diagram in mediation analysis.

Note: *a* coefficient effect of TyG on inflammatory indicator, X→M; *b* coefficient effect of inflammatory indicator on stage transition risk after controlling for TyG, M→Y; *c'* coefficient represents the direct effect of the independent variable X on the dependent variable Y after adjusting for inflammatory indicators X→Y. Abbreviations: TyG, triglyceride-glucose; 2hPG, 2-hour postprandial plasma glucose.

**Table S1.** Analysis results of the mediating effect of the TyG on inflammatory indicators between blood glucose

| X | M | Y | ACME | ADE | TE | CI_L | CI_H | *P* | *P*_adj | Sig | ACME absolute value |
|---|---|---|---|---|---|---|---|---|---|---|---|
| TyG | MLDLR | 2hPG | 0.0697 | 2.5746 | 2.6444 | 0.0191 | 0.1301 | 0.008 | 0.048 | Yes | 0.06972 |
| TyG | NLDLR | 2hPG | 0.0596 | 2.5818 | 2.6414 | 0.0156 | 0.1235 | 0.006 | 0.048 | Yes | 0.05955 |
| TyG | MHR | 2hPG | 0.0529 | 2.5884 | 2.6413 | -0.0108 | 0.1229 | 0.1 | 0.4 | No | 0.05291 |
| TyG | MLR | 2hPG | -0.0119 | 2.6508 | 2.6389 | -0.0413 | 0.0074 | 0.254 | 0.762 | No | 0.01192 |
| TyG | LLR | 2hPG | -0.0109 | 2.6455 | 2.6346 | -0.4435 | 0.3909 | 0.95 | 0.998 | No | 0.01093 |
| TyG | NHDLR | 2hPG | 0.0074 | 2.6399 | 2.6474 | -0.0484 | 0.0649 | 0.804 | 0.998 | No | 0.00743 |
| TyG | SII | 2hPG | -0.0023 | 2.6399 | 2.6376 | -0.0229 | 0.0142 | 0.774 | 0.998 | No | 0.00234 |
| TyG | NLR | 2hPG | -0.0007 | 2.6455 | 2.6448 | -0.0144 | 0.0132 | 0.96 | 0.998 | No | 0.00067 |
| TyG | LHR | 2hPG | 0.0002 | 2.6454 | 2.6456 | -0.0511 | 0.0515 | 0.952 | 0.998 | No | 0.00021 |
| TyG | AISI | 2hPG | 0.0002 | 2.6460 | 2.6462 | -0.0103 | 0.0124 | 0.998 | 0.998 | No | 0.00018 |
| TyG | SIRI | 2hPG | -0.0001 | 2.6439 | 2.6438 | -0.0126 | 0.0119 | 0.988 | 0.998 | No | 0.00012 |
| TyG | NMLR | 2hPG | 0.0000 | 2.6541 | 2.6541 | -0.0112 | 0.0115 | 0.972 | 0.998 | No | 0.00004 |

Note: ACME = Average Causal Mediation Effect; ADE = Average Direct Effect; TE = Total Effect; CI_L and CI_H = lower and upper 95% confidence intervals for ACME; *P* = original *P*-value for ACME; *P*_adj = *P*-value adjusted by Benjamini-Hochberg method; Sig = significant after BH adjustment (adjusted $P < 0.05$); ACME absolute value = absolute value of ACME. All variables were standardized prior to analysis. Abbreviations: TyG, triglyceride-glucose; 2hPG, 2-hour postprandial plasma glucose; T2DM, type 2 diabetes mellitus; AISI = (Neutrophil count × Platelet count × Monocyte count) / Lymphocyte count; SIRI = (Neutrophil count × Monocyte count) / Lymphocyte count; NMLR = (Monocyte count + Neutrophil count) / Lymphocyte count; MLR = (Monocyte count / Lymphocyte count); NLR = (Neutrophil count / Lymphocyte count); SII = (Platelet count × Neutrophil count) / Lymphocyte count; LHR = (Lymphocytes / HDL); LLR = (Lymphocytes * LDL); MHR = (Monocytes / HDL); MLDLR = (Monocytes * LDL); NLDLR = (Neutrophils * LDL); NHDLR = (Neutrophils/HDL).

**Table S2.** The significant mediating pathway between the TyG and blood glucose

| Index | MLDLR(2hPG) | NLDLR(2hPG) |
|---|---|---|
| ACME (mediating effect) | 0.0700 | 0.0596 |
| 95%CI | 0.0119 ~ 0.1300 | 0.0156 ~ 0.1236 |
| Original *P*-value | 0.008 | 0.006 |
| Correct *P*-value (BH) | 0.048 | 0.048 |
| *a* coefficient (TyG→M) | 0.080* | 0.101* |
| *b* coefficient (M→2hPG) | 0.128* | 0.085* |
| *c'* coefficient (TyG→2hPG) | 0.373* | 0.375* |

Note : ***$P<0.001$,**$P<0.01$,*$P<0.05$. Abbreviations: CI = confidence interval; BH =

Benjamini-Hochberg.)ACME, average causal mediation effect; TyG, triglyceride-glucose; 2hPG, 2-hour postprandial plasma glucose; MLDLR = (Monocytes * LDL); NLDLR = (Neutrophils * LDL).

**Table S3.** Comparison of key results between complete-case analysis (n=394) and multiply imputed dataset (n=1,117)

| Analysis | Complete-case analysis (n=394) | Multiple imputed dataset (n=1,117) | Consistency |
|---|---|---|---|
| Nonlinear association (GAM) | | | |
| edf (effective degrees of freedom) | 2.96 | 3.27 | Consistent |
| P-value for nonlinearity | $P < 0.001$ | $P < 0.001$ | Consistent |
| RCS inflection point | | | |
| TyG cut-off value | 9.14 | 9.14 | Consistent |
| Main effect direction | | | |
| TyG ↑ → T2DM risk ↑ | Positive | Positive | Consistent |
| TyG ↑ → 2hPG ↑ | Positive | Positive | Consistent |
| Mediation via MLDLR/NLDLR | | | |
| MLDLR indirect effect (95% CI) | −0.0089 (−0.0591, 0.0212) | 0.070 (0.019, 0.130) | See note |
| NLDLR indirect effect (95% CI) | 0.0160 (−0.0176, 0.0690) | 0.060 (0.016, 0.124) | See note |

**Table S4.** Complete results of the mediation effect for 394 full data cases

| X | M | Y | | Effect size | SE | CI | | Proportion of TE |
|---|---|---|---|---|---|---|---|---|
| | | | | | | CI_L | CI_H | |
| | | | TE | 1.1569 | | | | |
| TyG | NLR | 2hPG | DE | 1.1363 | 0.2897 | 0.5686 | 1.7041 | |
| | | | IE | 0.0206 | 0.0297 | -0.0340 | 0.0854 | 1.78% |
| | | | TE | 1.1281 | | | | |
| TyG | MLR | 2hPG | DE | 1.1248 | 0.2875 | 0.5614 | 1.6882 | |
| | | | IE | 0.0033 | 0.0215 | -0.0404 | 0.0489 | 0.29% |
| | | | TE | 1.1549 | | | | |
| TyG | NMLR | 2hPG | DE | 1.1351 | 0.2897 | 0.5672 | 1.7029 | |
| | | | IE | 0.0198 | 0.0297 | -0.0360 | 0.0864 | 1.71% |
| | | | TE | 1.1386 | | | | |
| TyG | SIRI | 2hPG | DE | 1.1379 | 0.2879 | 0.5737 | 1.7022 | |
| | | | IE | 0.0007 | 0.0199 | -0.0373 | 0.0470 | 0.06% |
| | | | TE | 1.1507 | | | | |
| TyG | SII | 2hPG | DE | 1.1172 | 0.2893 | 0.5501 | 1.6843 | |
| | | | IE | 0.0335 | 0.0313 | -0.0176 | 0.1073 | 2.91% |
| | | | TE | 1.1368 | | | | |
| TyG | AISI | 2hPG | DE | 1.1290 | 0.2880 | 0.5645 | 1.6935 | |
| | | | IE | 0.0078 | 0.0187 | -0.0306 | 0.0475 | 0.69% |
| TyG | LHR | 2hPG | TE | 1.0995 | | | | |

| X | M | Y | | Effect size | SE | CI | | Proportion of TE |
|---|---|---|---|---|---|---|---|---|
| | | | | | | CI_L | CI_H | |
| | | | DE | 1.1324 | 0.2861 | 0.5716 | 1.6932 | |
| | | | IE | -0.0329 | 0.1497 | -0.5011 | 0.0418 | 2.99% |
| | | | TE | 1.1237 | | | | |
| TyG | LLR | 2hPG | DE | 1.1383 | 0.2854 | 0.5790 | 1.6977 | |
| | | | IE | -0.0146 | 0.2905 | -0.9437 | 0.0214 | 1.30% |
| | | | TE | 1.1335 | | | | |
| TyG | MHR | 2hPG | DE | 1.1440 | 0.2859 | 0.5836 | 1.7044 | |
| | | | IE | -0.0105 | 0.0253 | -0.0670 | 0.0394 | 0.93% |
| | | | TE | 1.1344 | | | | |
| TyG | MLDLR | 2hPG | DE | 1.1433 | 0.2862 | 0.5823 | 1.7043 | |
| | | | IE | -0.0089 | 0.0198 | -0.0591 | 0.0212 | 0.78% |
| | | | TE | 1.1639 | | | | |
| TyG | NHDLR | 2hPG | DE | 1.1714 | 0.2879 | 0.6071 | 1.7357 | |
| | | | IE | -0.0075 | 0.0261 | -0.0601 | 0.0501 | 0.64% |
| | | | TE | 1.1535 | | | | |
| TyG | NLDLR | 2hPG | DE | 1.1375 | 0.2868 | 0.5755 | 1.6995 | |
| | | | IE | 0.0160 | 0.0218 | -0.0176 | 0.0690 | 1.39% |

Abbreviations: TE = Total Effect; DE = Direct Effect; IE = Indirect Effect; SE = Standard Error; CI_L and CI_H = lower and upper 95% confidence intervals for CI; AISI = (Neutrophil count × Platelet count × Monocyte count) / Lymphocyte count; SIRI = (Neutrophil count × Monocyte count) / Lymphocyte count; NMLR = (Monocyte count + Neutrophil count) / Lymphocyte count; MLR = (Monocyte count / Lymphocyte count); NLR = (Neutrophil count / Lymphocyte count); SII = (Platelet count × Neutrophil count) / Lymphocyte count; LHR = (Lymphocytes / HDL); LLR = (Lymphocytes * LDL); MHR = (Monocytes / HDL); MLDLR = (Monocytes * LDL); NLDLR = (Neutrophils * LDL); NHDLR = (Neutrophils / HDL).
